# 2-[4-*tert*-Butyl-5-(2-chloro­benz­yl)-1,3-thia­zol-2-yl]isoindoline-1,3-dione

**DOI:** 10.1107/S1600536810042601

**Published:** 2010-10-23

**Authors:** Zhi-Gang Yao, Jun-Mei Peng, Su-Fang Huo, Ai-Xi Hu

**Affiliations:** a School of Chemistry and Chemical Engineering, South China University of Technology, Guangzhou 510640, People’s Republic of China; b College of Chemistry and Chemical Engineering, Hunan University, Changsha 410082, People’s Republic of China

## Abstract

In the title compound, C_22_H_19_ClN_2_O_2_S, the dihedral angle between the phenyl­ene ring and the phthalimide ring system is 4.4 (1)°. There is no hydrogen bonding or π–π stacking in the crystal structure.

## Related literature

For background to thia­zole derivatives, see: Kazzouli *et al.* (2002 ▶); Holla *et al.* (2003 ▶); Hu *et al.* (2008 ▶), For background to phthalimide derivatives, see: Lima *et al.* (2002 ▶); Miyachi *et al.* (1997 ▶); Yachide *et al.* (2007 ▶).
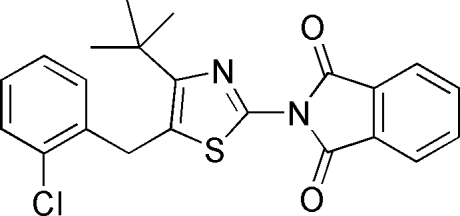


## Experimental

### 

#### Crystal data

C_22_H_19_ClN_2_O_2_S
*M* *_r_* = 410.90
Triclinic, 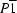

*a* = 7.8357 (4) Å
*b* = 8.1587 (4) Å
*c* = 16.1487 (8) Å
α = 100.404 (1)°
β = 95.897 (1)°
γ = 96.490 (1)°
*V* = 1000.85 (9) Å^3^
*Z* = 2
Mo *K*α radiation
μ = 0.32 mm^−1^
*T* = 173 K
0.46 × 0.30 × 0.28 mm

#### Data collection

Bruker SMART 1000 CCD diffractometer
Absorption correction: multi-scan (*SADABS*; Sheldrick, 2004 ▶) *T*_min_ = 0.868, *T*_max_ = 0.917
7798 measured reflections
3857 independent reflections
3268 reflections with *I* > 2σ(*I*)
*R*_int_ = 0.018

#### Refinement

*R*[*F* ^2^ > 2σ(*F* ^2^)] = 0.036
*wR*(*F* ^2^) = 0.122
*S* = 1.15
3857 reflections
256 parameters
H-atom parameters constrained
Δρ_max_ = 0.33 e Å^−3^
Δρ_min_ = −0.31 e Å^−3^

### 

Data collection: *SMART* (Bruker, 2001 ▶); cell refinement: *SAINT-Plus* (Bruker, 2003 ▶); data reduction: *SAINT-Plus*; program(s) used to solve structure: *SHELXS97* (Sheldrick, 2008 ▶); program(s) used to refine structure: *SHELXL97* (Sheldrick, 2008 ▶); molecular graphics: *SHELXTL* (Sheldrick, 2008 ▶); software used to prepare material for publication: *SHELXTL*.

## Supplementary Material

Crystal structure: contains datablocks I, global. DOI: 10.1107/S1600536810042601/ng5049sup1.cif

Structure factors: contains datablocks I. DOI: 10.1107/S1600536810042601/ng5049Isup2.hkl

Additional supplementary materials:  crystallographic information; 3D view; checkCIF report

## Figures and Tables

**Fig. 1.**
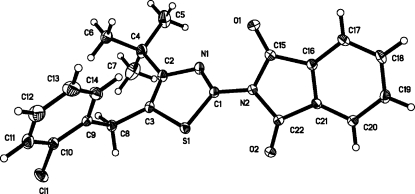
The molecular structure of the title compound, showing the atom-labelling scheme and 50% probability displacement ellipsoid (arbitrary spheres for H atoms).

**Fig. 2.**
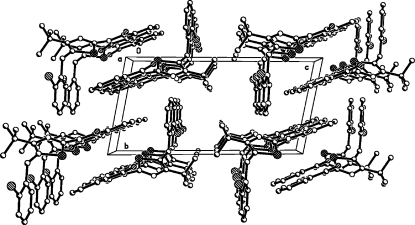
A packing diagram for the title compound.

## supplementary crystallographic information

### Comment

Compounds containing thiazole ring have a wide spectrum of biological activities, many of them are well known as antiviral, antifungal agents (Kazzouli *et al.*, 2002; Holla *et al.*, 2003; Hu *et al.*, 2008). *N*-substituted phthalimide derivatives are very important in pharmaceutical intermediates and drugs (Miyachi *et al.*, 1997). Herein we report the synthesis and the crystal structure of the phthalimide compounds which contain the thiazole ring.

The molecular structure of the title compound, C_22_H_19_ClN_2_O_2_S, is shown in Fig 1. There are no hydrogen bonding and π–π stacking in the crystal structure. The van der Waals interactions maintain the structural cohesion.

### Experimental

A solution of 10 mmol 5-(2-chlorobenzyl)-4-*tert*-butylthiazol-2-amine and 10 mmol ph thalic anhydride in 15 ml acetic acid, then heated and refluxed for 23 h. After finishing the reaction, cooled the solution, and the precipitate formed, filtered, recrystallized with ethanol to give the title compound. The crystals for X-ray structure determination were obtained by slow evaporation of an ethanol solution at room temperature.

### Refinement

The crystal system of the title compound was triclinic and all H atoms were refined using riding mode. The C—H distances of phenyl and *tert*-butyl were 0.95Å and 0.98 Å, with *U*_iso_(*H*)=1.5*U*_eq_(C_methyl_).

### Crystal data

**Table:**

C_22_H_19_ClN_2_O_2_S	*Z* = 2
*M**_r_* = 410.90	*F*(000) = 428
Triclinic, *P*1	*D*_x_ = 1.363 Mg m^−^^3^
Hall symbol: -P 1	Melting point: 425 K
*a* = 7.8357 (4) Å	Mo *K*α radiation, λ = 0.71073 Å
*b* = 8.1587 (4) Å	Cell parameters from 5125 reflections
*c* = 16.1487 (8) Å	θ = 2.6–27.0°
α = 100.404 (1)°	µ = 0.32 mm^−^^1^
β = 95.897 (1)°	*T* = 173 K
γ = 96.490 (1)°	Block, colorless
*V* = 1000.85 (9) Å^3^	0.46 × 0.30 × 0.28 mm

### Data collection

**Table:**

Bruker SMART 1000 CCD diffractometer	3857 independent reflections
Radiation source: fine-focus sealed tube	3268 reflections with *I* > 2σ(*I*)
graphite	*R*_int_ = 0.018
ω scans	θ_max_ = 26.0°, θ_min_ = 2.6°
Absorption correction: multi-scan (*SADABS*; Sheldrick, 2004)	*h* = −9→9
*T*_min_ = 0.868, *T*_max_ = 0.917	*k* = −10→10
7798 measured reflections	*l* = −19→19

### Refinement

**Table:**

Refinement on *F*^2^	Primary atom site location: structure-invariant direct methods
Least-squares matrix: full	Secondary atom site location: difference Fourier map
*R*[*F*^2^ > 2σ(*F*^2^)] = 0.036	Hydrogen site location: inferred from neighbouring sites
*wR*(*F*^2^) = 0.122	H-atom parameters constrained
*S* = 1.15	*w* = 1/[σ^2^(*F*_o_^2^) + (0.0649*P*)^2^ + 0.4002*P*] where *P* = (*F*_o_^2^ + 2*F*_c_^2^)/3
3857 reflections	(Δ/σ)_max_ < 0.001
256 parameters	Δρ_max_ = 0.33 e Å^−^^3^
0 restraints	Δρ_min_ = −0.31 e Å^−^^3^

### Special details

**Table:**

Experimental. The ^1^HNMR(CDCl_3_, 400 MHz) of the title compound were: 1.46(s,9*H*,3×CH_3_), 4.41 (s, 2H, CH_2_), 7.18~7.39 (m, 4H, C_6_H_4_), 7.79~7.96 (m, 4H, C_6_H_4_).And the yield was: 67.6%. m.p.423~427 K.
Geometry. All e.s.d.'s (except the e.s.d. in the dihedral angle between two l.s. planes) are estimated using the full covariance matrix. The cell e.s.d.'s are taken into account individually in the estimation of e.s.d.'s in distances, angles and torsion angles; correlations between e.s.d.'s in cell parameters are only used when they are defined by crystal symmetry. An approximate (isotropic) treatment of cell e.s.d.'s is used for estimating e.s.d.'s involving l.s. planes.
Refinement. Refinement of *F*^2^ against ALL reflections. The weighted *R*-factor *wR* and goodness of fit *S* are based on *F*^2^, conventional *R*-factors *R* are based on *F*, with *F* set to zero for negative *F*^2^. The threshold expression of *F*^2^ > σ(*F*^2^) is used only for calculating *R*-factors(gt) *etc*. and is not relevant to the choice of reflections for refinement. *R*-factors based on *F*^2^ are statistically about twice as large as those based on *F*, and *R*- factors based on ALL data will be even larger.

### Fractional atomic coordinates and isotropic or equivalent isotropic displacement parameters (Å^2^)

**Table:**

	*x*	*y*	*z*	*U*_iso_*/*U*_eq_	
Cl1	−0.47492 (7)	−0.21014 (8)	0.32706 (3)	0.04128 (18)	
S1	0.02664 (6)	0.03724 (6)	0.16354 (3)	0.02634 (15)	
C1	0.2305 (2)	0.1497 (2)	0.18229 (12)	0.0236 (4)	
C2	0.1795 (3)	0.1461 (2)	0.31377 (12)	0.0266 (4)	
C3	0.0266 (2)	0.0574 (2)	0.27220 (12)	0.0239 (4)	
C4	0.2332 (3)	0.1918 (3)	0.40993 (13)	0.0366 (5)	
C5	0.4226 (4)	0.2725 (4)	0.42868 (16)	0.0624 (9)	
H5A	0.4953	0.1967	0.3993	0.094*	
H5B	0.4592	0.2932	0.4900	0.094*	
H5C	0.4347	0.3793	0.4088	0.094*	
C6	0.2175 (3)	0.0330 (3)	0.44847 (14)	0.0389 (5)	
H6A	0.0978	−0.0232	0.4360	0.058*	
H6B	0.2495	0.0640	0.5101	0.058*	
H6C	0.2950	−0.0434	0.4240	0.058*	
C7	0.1182 (4)	0.3151 (3)	0.45113 (15)	0.0555 (7)	
H7A	0.1307	0.4177	0.4275	0.083*	
H7B	0.1534	0.3433	0.5126	0.083*	
H7C	−0.0029	0.2630	0.4396	0.083*	
C8	−0.1313 (2)	−0.0235 (2)	0.30286 (12)	0.0261 (4)	
H8A	−0.2359	0.0057	0.2720	0.031*	
H8B	−0.1298	0.0241	0.3639	0.031*	
C9	−0.1445 (2)	−0.2135 (2)	0.29113 (12)	0.0255 (4)	
C10	−0.2985 (3)	−0.3103 (3)	0.29935 (12)	0.0298 (4)	
C11	−0.3166 (3)	−0.4842 (3)	0.28567 (15)	0.0422 (6)	
H11	−0.4235	−0.5467	0.2909	0.051*	
C12	−0.1785 (4)	−0.5656 (3)	0.26456 (18)	0.0510 (7)	
H12	−0.1901	−0.6849	0.2547	0.061*	
C13	−0.0231 (3)	−0.4749 (3)	0.25766 (18)	0.0492 (6)	
H13	0.0730	−0.5313	0.2441	0.059*	
C14	−0.0074 (3)	−0.3009 (3)	0.27052 (15)	0.0349 (5)	
H14	0.1001	−0.2396	0.2651	0.042*	
C15	0.4999 (2)	0.1549 (2)	0.11270 (12)	0.0248 (4)	
C16	0.5493 (2)	0.2158 (2)	0.03643 (12)	0.0245 (4)	
C17	0.7093 (3)	0.2341 (3)	0.00803 (14)	0.0306 (4)	
H17	0.8079	0.2015	0.0369	0.037*	
C18	0.7203 (3)	0.3020 (3)	−0.06437 (13)	0.0319 (5)	
H18	0.8292	0.3190	−0.0847	0.038*	
C19	0.5758 (3)	0.3453 (3)	−0.10749 (13)	0.0307 (4)	
H19	0.5873	0.3896	−0.1574	0.037*	
C20	0.4131 (2)	0.3257 (3)	−0.07953 (12)	0.0270 (4)	
H20	0.3135	0.3546	−0.1094	0.032*	
C21	0.4048 (2)	0.2618 (2)	−0.00597 (11)	0.0228 (4)	
C22	0.2572 (2)	0.2381 (2)	0.04291 (12)	0.0249 (4)	
N1	0.2936 (2)	0.2002 (2)	0.26111 (10)	0.0276 (4)	
N2	0.3239 (2)	0.1807 (2)	0.11564 (10)	0.0244 (3)	
O1	0.58277 (18)	0.09357 (19)	0.16259 (9)	0.0334 (3)	
O2	0.10930 (18)	0.2615 (2)	0.02800 (9)	0.0374 (4)	

### Atomic displacement parameters (Å^2^)

**Table:**

	*U*^11^	*U*^22^	*U*^33^	*U*^12^	*U*^13^	*U*^23^
Cl1	0.0275 (3)	0.0591 (4)	0.0341 (3)	−0.0019 (2)	0.0104 (2)	0.0021 (2)
S1	0.0230 (3)	0.0335 (3)	0.0218 (3)	−0.00074 (19)	0.00394 (18)	0.00557 (19)
C1	0.0242 (9)	0.0245 (9)	0.0229 (9)	0.0027 (7)	0.0047 (7)	0.0062 (7)
C2	0.0319 (10)	0.0250 (10)	0.0225 (10)	0.0002 (8)	0.0058 (8)	0.0043 (7)
C3	0.0285 (10)	0.0230 (9)	0.0224 (9)	0.0048 (7)	0.0070 (8)	0.0071 (7)
C4	0.0447 (13)	0.0408 (12)	0.0211 (10)	−0.0054 (10)	0.0049 (9)	0.0044 (9)
C5	0.0642 (18)	0.083 (2)	0.0264 (12)	−0.0353 (15)	−0.0045 (12)	0.0075 (12)
C6	0.0412 (13)	0.0510 (14)	0.0266 (11)	0.0052 (10)	0.0068 (9)	0.0125 (10)
C7	0.092 (2)	0.0431 (14)	0.0298 (12)	0.0145 (14)	0.0097 (13)	−0.0021 (10)
C8	0.0262 (10)	0.0280 (10)	0.0266 (10)	0.0047 (8)	0.0073 (8)	0.0086 (8)
C9	0.0261 (10)	0.0284 (10)	0.0225 (9)	0.0018 (8)	0.0003 (7)	0.0093 (8)
C10	0.0302 (10)	0.0370 (11)	0.0217 (9)	−0.0027 (8)	0.0026 (8)	0.0090 (8)
C11	0.0456 (13)	0.0381 (13)	0.0398 (13)	−0.0132 (10)	−0.0033 (10)	0.0154 (10)
C12	0.0573 (16)	0.0255 (11)	0.0673 (17)	−0.0004 (11)	−0.0070 (13)	0.0134 (11)
C13	0.0443 (14)	0.0326 (12)	0.0706 (18)	0.0120 (10)	−0.0009 (12)	0.0105 (12)
C14	0.0297 (11)	0.0294 (11)	0.0465 (13)	0.0037 (8)	0.0038 (9)	0.0105 (9)
C15	0.0214 (9)	0.0261 (10)	0.0266 (10)	0.0022 (7)	0.0031 (7)	0.0050 (8)
C16	0.0234 (9)	0.0258 (10)	0.0242 (9)	0.0023 (7)	0.0047 (7)	0.0044 (8)
C17	0.0198 (9)	0.0369 (11)	0.0366 (11)	0.0055 (8)	0.0064 (8)	0.0086 (9)
C18	0.0247 (10)	0.0372 (11)	0.0350 (11)	0.0021 (8)	0.0135 (9)	0.0060 (9)
C19	0.0345 (11)	0.0343 (11)	0.0227 (10)	−0.0003 (8)	0.0078 (8)	0.0045 (8)
C20	0.0244 (10)	0.0340 (11)	0.0227 (9)	0.0033 (8)	0.0019 (8)	0.0062 (8)
C21	0.0197 (9)	0.0260 (10)	0.0213 (9)	0.0006 (7)	0.0036 (7)	0.0023 (7)
C22	0.0232 (10)	0.0290 (10)	0.0227 (9)	0.0022 (7)	0.0016 (7)	0.0068 (8)
N1	0.0315 (9)	0.0268 (8)	0.0237 (8)	−0.0020 (7)	0.0060 (7)	0.0052 (7)
N2	0.0205 (8)	0.0309 (9)	0.0236 (8)	0.0034 (6)	0.0039 (6)	0.0093 (6)
O1	0.0284 (7)	0.0411 (8)	0.0343 (8)	0.0081 (6)	0.0003 (6)	0.0166 (7)
O2	0.0208 (7)	0.0607 (10)	0.0362 (8)	0.0081 (7)	0.0049 (6)	0.0216 (7)

### Geometric parameters (Å, °)

**Table:**

Cl1—C10	1.741 (2)	C9—C10	1.397 (3)
S1—C1	1.7188 (19)	C10—C11	1.385 (3)
S1—C3	1.7327 (19)	C11—C12	1.373 (4)
C1—N1	1.292 (2)	C11—H11	0.9500
C1—N2	1.406 (2)	C12—C13	1.377 (4)
C2—C3	1.369 (3)	C12—H12	0.9500
C2—N1	1.385 (2)	C13—C14	1.386 (3)
C2—C4	1.532 (3)	C13—H13	0.9500
C3—C8	1.507 (3)	C14—H14	0.9500
C4—C5	1.531 (3)	C15—O1	1.197 (2)
C4—C6	1.534 (3)	C15—N2	1.422 (2)
C4—C7	1.534 (3)	C15—C16	1.480 (3)
C5—H5A	0.9800	C16—C17	1.380 (3)
C5—H5B	0.9800	C16—C21	1.386 (3)
C5—H5C	0.9800	C17—C18	1.388 (3)
C6—H6A	0.9800	C17—H17	0.9500
C6—H6B	0.9800	C18—C19	1.382 (3)
C6—H6C	0.9800	C18—H18	0.9500
C7—H7A	0.9800	C19—C20	1.398 (3)
C7—H7B	0.9800	C19—H19	0.9500
C7—H7C	0.9800	C20—C21	1.385 (3)
C8—C9	1.518 (3)	C20—H20	0.9500
C8—H8A	0.9900	C21—C22	1.480 (3)
C8—H8B	0.9900	C22—O2	1.203 (2)
C9—C14	1.391 (3)	C22—N2	1.414 (2)
			
C1—S1—C3	88.77 (9)	C11—C10—C9	122.3 (2)
N1—C1—N2	122.33 (17)	C11—C10—Cl1	118.45 (17)
N1—C1—S1	115.89 (14)	C9—C10—Cl1	119.27 (16)
N2—C1—S1	121.76 (14)	C12—C11—C10	119.4 (2)
C3—C2—N1	114.54 (17)	C12—C11—H11	120.3
C3—C2—C4	127.00 (18)	C10—C11—H11	120.3
N1—C2—C4	118.45 (17)	C11—C12—C13	120.3 (2)
C2—C3—C8	132.65 (17)	C11—C12—H12	119.9
C2—C3—S1	109.93 (14)	C13—C12—H12	119.9
C8—C3—S1	117.41 (14)	C12—C13—C14	119.8 (2)
C5—C4—C2	109.70 (17)	C12—C13—H13	120.1
C5—C4—C6	107.7 (2)	C14—C13—H13	120.1
C2—C4—C6	110.24 (18)	C13—C14—C9	121.9 (2)
C5—C4—C7	109.5 (2)	C13—C14—H14	119.1
C2—C4—C7	110.09 (19)	C9—C14—H14	119.1
C6—C4—C7	109.56 (19)	O1—C15—N2	124.78 (18)
C4—C5—H5A	109.5	O1—C15—C16	129.95 (18)
C4—C5—H5B	109.5	N2—C15—C16	105.26 (15)
H5A—C5—H5B	109.5	C17—C16—C21	121.50 (18)
C4—C5—H5C	109.5	C17—C16—C15	129.50 (18)
H5A—C5—H5C	109.5	C21—C16—C15	108.97 (16)
H5B—C5—H5C	109.5	C16—C17—C18	117.32 (18)
C4—C6—H6A	109.5	C16—C17—H17	121.3
C4—C6—H6B	109.5	C18—C17—H17	121.3
H6A—C6—H6B	109.5	C19—C18—C17	121.24 (18)
C4—C6—H6C	109.5	C19—C18—H18	119.4
H6A—C6—H6C	109.5	C17—C18—H18	119.4
H6B—C6—H6C	109.5	C18—C19—C20	121.71 (19)
C4—C7—H7A	109.5	C18—C19—H19	119.1
C4—C7—H7B	109.5	C20—C19—H19	119.1
H7A—C7—H7B	109.5	C21—C20—C19	116.44 (18)
C4—C7—H7C	109.5	C21—C20—H20	121.8
H7A—C7—H7C	109.5	C19—C20—H20	121.8
H7B—C7—H7C	109.5	C20—C21—C16	121.75 (17)
C3—C8—C9	113.97 (16)	C20—C21—C22	129.59 (17)
C3—C8—H8A	108.8	C16—C21—C22	108.59 (16)
C9—C8—H8A	108.8	O2—C22—N2	124.31 (18)
C3—C8—H8B	108.8	O2—C22—C21	129.93 (18)
C9—C8—H8B	108.8	N2—C22—C21	105.75 (15)
H8A—C8—H8B	107.7	C1—N1—C2	110.85 (16)
C14—C9—C10	116.45 (19)	C1—N2—C22	125.37 (16)
C14—C9—C8	122.59 (17)	C1—N2—C15	123.47 (15)
C10—C9—C8	120.96 (18)	C22—N2—C15	111.15 (15)
			
C3—S1—C1—N1	−0.76 (16)	N2—C15—C16—C21	−4.5 (2)
C3—S1—C1—N2	177.63 (16)	C21—C16—C17—C18	0.6 (3)
N1—C2—C3—C8	−179.96 (18)	C15—C16—C17—C18	−177.14 (19)
C4—C2—C3—C8	−0.9 (4)	C16—C17—C18—C19	−1.7 (3)
N1—C2—C3—S1	1.1 (2)	C17—C18—C19—C20	1.1 (3)
C4—C2—C3—S1	−179.79 (17)	C18—C19—C20—C21	0.5 (3)
C1—S1—C3—C2	−0.25 (15)	C19—C20—C21—C16	−1.6 (3)
C1—S1—C3—C8	−179.33 (15)	C19—C20—C21—C22	175.01 (18)
C3—C2—C4—C5	173.1 (2)	C17—C16—C21—C20	1.1 (3)
N1—C2—C4—C5	−7.9 (3)	C15—C16—C21—C20	179.23 (17)
C3—C2—C4—C6	54.6 (3)	C17—C16—C21—C22	−176.19 (18)
N1—C2—C4—C6	−126.4 (2)	C15—C16—C21—C22	2.0 (2)
C3—C2—C4—C7	−66.4 (3)	C20—C21—C22—O2	3.8 (4)
N1—C2—C4—C7	112.6 (2)	C16—C21—C22—O2	−179.2 (2)
C2—C3—C8—C9	−104.1 (2)	C20—C21—C22—N2	−175.65 (19)
S1—C3—C8—C9	74.74 (19)	C16—C21—C22—N2	1.3 (2)
C3—C8—C9—C14	12.5 (3)	N2—C1—N1—C2	−176.86 (17)
C3—C8—C9—C10	−166.43 (18)	S1—C1—N1—C2	1.5 (2)
C14—C9—C10—C11	−1.7 (3)	C3—C2—N1—C1	−1.7 (2)
C8—C9—C10—C11	177.34 (19)	C4—C2—N1—C1	179.14 (18)
C14—C9—C10—Cl1	178.77 (15)	N1—C1—N2—C22	−134.9 (2)
C8—C9—C10—Cl1	−2.2 (3)	S1—C1—N2—C22	46.8 (2)
C9—C10—C11—C12	1.0 (3)	N1—C1—N2—C15	46.1 (3)
Cl1—C10—C11—C12	−179.38 (19)	S1—C1—N2—C15	−132.20 (17)
C10—C11—C12—C13	0.5 (4)	O2—C22—N2—C1	−2.9 (3)
C11—C12—C13—C14	−1.2 (4)	C21—C22—N2—C1	176.62 (16)
C12—C13—C14—C9	0.6 (4)	O2—C22—N2—C15	176.22 (19)
C10—C9—C14—C13	0.8 (3)	C21—C22—N2—C15	−4.3 (2)
C8—C9—C14—C13	−178.1 (2)	O1—C15—N2—C1	5.6 (3)
O1—C15—C16—C17	−7.7 (4)	C16—C15—N2—C1	−175.46 (16)
N2—C15—C16—C17	173.5 (2)	O1—C15—N2—C22	−173.47 (19)
O1—C15—C16—C21	174.3 (2)	C16—C15—N2—C22	5.4 (2)
